# Supplementary material for: Network-wide thermodynamic constraints shape NAD(P)H cofactor specificity of biochemical reactions
Source: Nat Commun. 2023 Aug 3;14:4660. doi: 10.1038/s41467-023-40297-8 (PMC10400544; doi:10.1038/s41467-023-40297-8)
Supplement: Supplementary file 1 — Supplementary Information [file 41467_2023_40297_MOESM1_ESM.pdf]

Supplementary Information

Network-wide Thermodynamic Constraints Shape NAD(P)H Cofactor Specificity of Biochemical Reactions

Pavlos Stephanos Bekiaris[1] and Steffen Klamt[1,*]

[1]Max Planck Institute for Dynamics of Complex Technical Systems, Magdeburg, Sandtorstr. 1, Germany

*Corresponding author: klamt@mpi-magdeburg.mpg.de

Supplementary Note 1: Removal of thermodynamic bottlenecks to make iML1515 thermodynamically feasible

With the used $\Delta_r G'^{\circ}$ values and standard metabolite concentration ranges (see Methods section in the main text), we found that growth in the iML1515 model is thermodynamically infeasible under aerobic as well as anaerobic conditions as the determined MDF is very negative (ca. -4705 kJ/mol) for both conditions. We used the following algorithm to resolve this infeasibility by relaxing a minimal set of thermodynamic constraints to enable growth (with 99% of the maximal growth rate, see Methods section in the main text) with an MDF of at least 0.1 kJ/mol.

As already described in the original MDF[1] and the generalized OptMDFpathway[2] formulations, MDF-optimal solutions contain always one or more bottleneck reactions limiting the MDF. Formally, bottleneck reactions are those where an decrease in their standard Gibbs free energy $\Delta_r G'^{\circ}$ increases the MDF[2]. Bottleneck reactions can be identified as follows: we introduce a new set of binary variables $z_i^b \in \{0,1\}$ for all reactions and modify Equation (7) to

$$B \leq f_i + M \cdot (1 - z_i) + M \cdot z_i^b \quad \text{(S1).}$$

For each reaction i, one after the other, we could set $z_i^b = 1$ (which practically removes the driving force constraint for this reaction) and $z_j^b = 0$ for all other reactions $(j \neq i)$ and recompute the MDF via Equation (9) or Equation (12), respectively. If the previously found MDF value increases, then reaction i is a bottleneck. A similar setting can now be used to identify a minimal number of reactions, which, if their thermodynamic constraint is relaxed, make a thermodynamically infeasible system feasible. For this we demand a minimum network-wide MDF of 0.1 kJ/mol ($B \geq B_{min} = 0.1$), leave all z_i^b unconstrained and minimize the sum of all relaxations:

$$\underset{x,r}{Minimize} \ \sum z_i^b$$

$s.t. eqs.$ $(1) - (8)$, $(S1)$, and (14) or (15).

Our bottleneck-removing procedure has the same goal as the “relax_dgo” routine of pyTFA [3], although the latter minimizes the sum of all $\Delta_r G'^{\circ}$changes while our method minimizes the number of reactions whose $\Delta_r G'^{\circ}$must be decreased.

Applying this algorithm led to the identification of 9 reactions (Supplementary Table 1) whose thermodynamic constraints were relaxed (by setting their $\Delta_r G'^{\circ}$ to -100 kJ/mol) to enable growth with the respective maximal growth rate under aerobic and anaerobic conditions. The identified bottleneck reactions occur only in anabolic pathways, requiring small yet essential fluxes, and their $\Delta_r G'^{\circ}$ values are often very high (up to a maximum of 4125 kJ/mol) indicating potentially unrealistic values.

Supplementary Table 1: List of reactions with relaxed $\Delta_r G'^{\circ}$ values to enable growth in the iML1515_TCOSA model. *Note that the $\Delta_r G'^{\circ}$ of the AIRC3 reaction was changed to +100 kJ to enable the reverse direction required for growth. All reactions except AIRC3 are irreversible.*

**Reaction BiGG ID**	**Name of the enzyme catalyzing the reaction**	**$\Delta_r G'^{\circ}$ [kJ/mol] as calculated by eQuilibrator**	**Corrected (relaxed) $\Delta_r G'^{\circ}$ [kJ/mol]**
KDOCT2	3-deoxy-manno-octulosonate cytidylyltransferase	+225.43	-100
MECDPS	2-C-methyl-D-erythritol 2,4-cyclodiphosphate synthase	+1809.64	-100
DHPPDA2	Diaminohydroxyphosphoribosylaminopryrimidine deaminase	+278.17	-100
ATPPRT	ATP phosphoribosyltransferase	+4125.05	-100
IG3PS	Imidazole-glycerol-3-phosphate synthase	+233.36	-100
MCTP1App	Murein crosslinking transpeptidase	+269.73	-100
MALCOAMT	Malonyl-CoA methyltransferase	+55.9	-100
AIRC3	Phosphoribosylaminoimidazole carboxylase	-30.08	+100
SHCHD2	Sirohydrochlorin dehydrogenase	+2432.76	-100

Supplementary Note 2: Results with sampled $\Delta_r G'^\circ$ values

To analyze the uncertainty of our thermodynamic calculations with respect to the used (estimated) $\Delta_r G'^\circ$ values, we re-performed several calculations with sampled $\Delta_r G'^\circ$ values (with glucose as substrate). Sampling of the $\Delta_r G'^\circ$ values was set up as follows. In order to keep thermodynamic consistency throughout changing the $\Delta_r G'^\circ$ (cf. Beber et al.[4]), we added a random value to the $\Delta_f G'^\circ$ (standard Gibbs free energies of formation) of all metabolites. The relationship between the $\Delta_f \boldsymbol{G}'^\circ$ and the $\Delta_r \boldsymbol{G}'^\circ$ (from here, both are considered as vectors written in bold) reads

$$\Delta_r \boldsymbol{G}'^\circ = \mathbf{N}^T \Delta_f \boldsymbol{G}'^\circ.$$

Hence, even without knowledge of the $\Delta_f \boldsymbol{G}'^\circ$, we can, in a thermodynamically consistent manner, obtain varied $\Delta_r \boldsymbol{G}'^\circ$ values (denoted by $\Delta_r \widehat{\boldsymbol{G}}'^\circ$) by adding random values $\boldsymbol{\delta}$ to the (possibly unknown) $\Delta_f \boldsymbol{G}'^\circ$:

$$\Delta_r \widehat{\boldsymbol{G}}'^\circ = \mathbf{N}^{\boldsymbol{T}}(\Delta_f \boldsymbol{G}'^\circ + \boldsymbol{\delta}) = \mathbf{N}^{\boldsymbol{T}} \Delta_f \boldsymbol{G}'^\circ + \mathbf{N}^{\boldsymbol{T}} \boldsymbol{\delta} = \Delta_r \boldsymbol{G}'^\circ + \mathbf{N}^{\boldsymbol{T}} \boldsymbol{\delta}.$$

The values in $\boldsymbol{\delta}$ were uniformly sampled in the range from -5 kJ/mol to +5 kJ/mol. It should be noted that the resulting variation in the $\Delta_r \boldsymbol{G}'^\circ$ can be larger, for example, for a reaction A + B → C + D it can be up to ± 20 kJ/mol. As the only exceptions, the $\Delta_f G'^\circ$ values of the ubiquitous small molecules H^+, H_2O, O_2 and CO_2 were not changed. In this way, we created 100 sampled $\Delta_r G'^\circ$ sets, for all of which we calculated the resulting (Sub)MDF values for the single-cofactor, wild-type and flexible specificity scenario.

Supplementary Fig. 1 shows the results obtained with the randomly sampled $\Delta_r G'^\circ$ values. Analogous to Figure 3 in the main text, it can clearly be seen that, under all conditions, the mean (Sub)MDF values for the wild-type specificity are still close or even identical to the mean (Sub)MDF values obtainable with the flexible specificity and it is significantly higher than for the single cofactor specificity scenario. Interestingly, for all specificities, the mean (Sub)MDF values for the sampled $\Delta_r G'^\circ$ values are almost always lower than the original values. This may be caused by the high likelihood that an increase of the $\Delta_r G'^\circ$ value of any (stoichiometrically) essential reaction may create a new thermodynamic bottleneck. That the $\Delta_r G'^\circ$ value of all bottleneck reactions of the original model are decreased (relaxed) simultaneously is less likely.

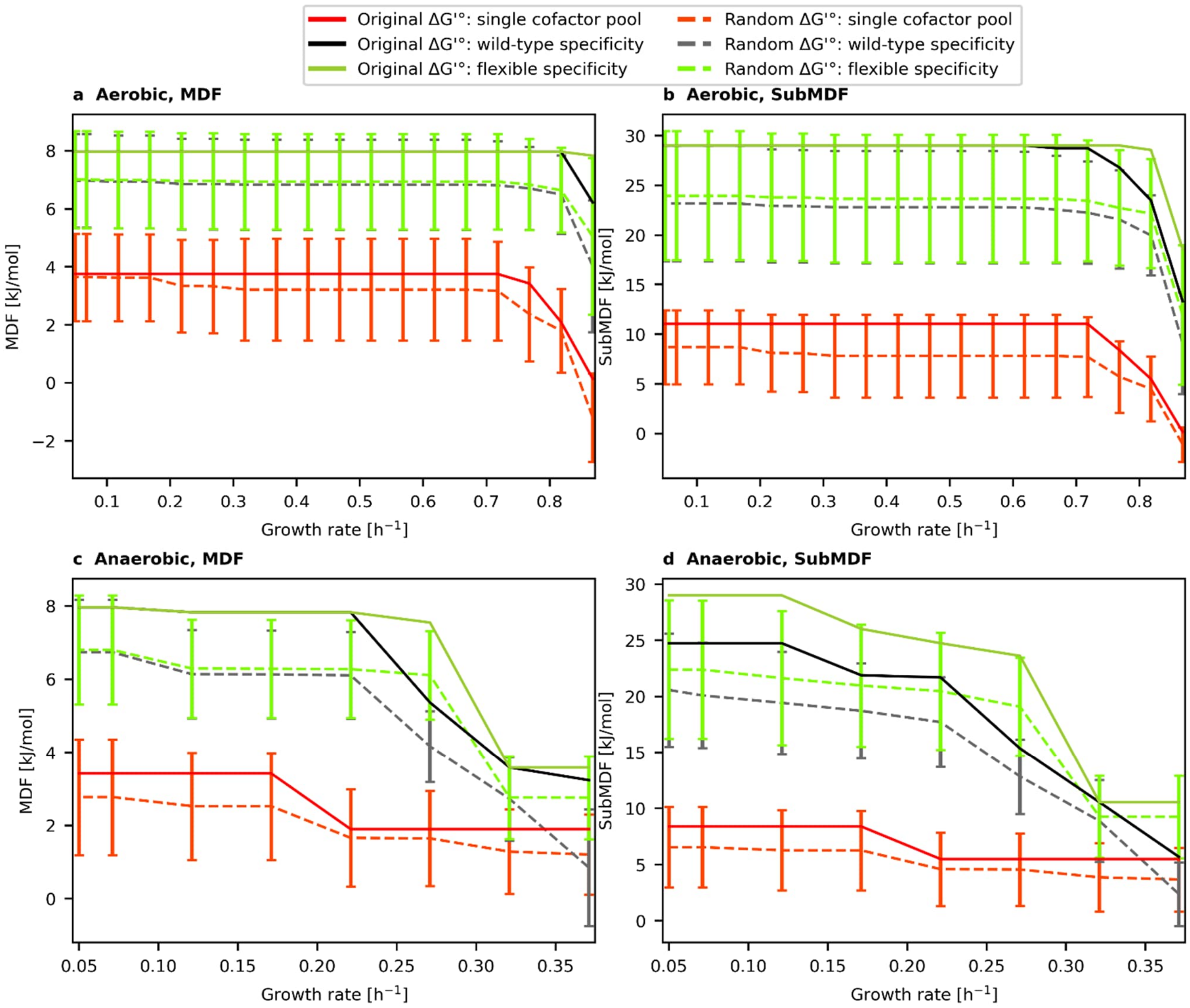

Supplementary Figure 1. **Comparison of (Sub)MDF results for 100 sampled $\Delta_r G'^{\circ}$ values (dashed line) with the originally used $\Delta_r G'^{\circ}$ values (taken from Fig. 2 in the main text).** ***a*** *Aerobic conditions with MDF as optimization target.* ***b*** *Aerobic conditions with SubMDF as target.* ***c*** *Anaerobic conditions with MDF as target.* ***d*** *Anaerobic conditions with SubMDF as target. The data points on the dashed lines represent the mean value of all 100 tested random sets of $\Delta_r G'^{\circ}$ values and the error bars indicate the standard deviation. Source data are provided as a Source Data file.*

Supplementary Note 3: Results with tight (measured) concentration ranges

To analyze the sensitivity of our results with respect to the metabolite concentrations, we re-performed several analyses also with much tighter metabolite concentration ranges (with glucose as substrate). The latter were taken from reported metabolomic data for *E. coli* growing aerobically on glucose[5]. If the concentration of a metabolite contained in the model was measured in Bennett et al.[5], then the upper and lower bound for the concentration of this metabolite was constrained according to the given 95% confidence interval of the measured concentration. For metabolites where Bennett et al.[5] did not report measurements, the standard concentrations ranges (see Methods) were used.

As for the standard concentration ranges, the $\Delta_r G'^\circ$ values of some reactions had to be relaxed to make the model thermodynamically feasible (see Supplementary Note 1). With the tighter (measured) concentration ranges we found that the $\Delta_r G'^\circ$ values of the same 9 reactions as found for the standard concentrations (Supplementary Table 1) plus three additional reactions (Supplementary Table 2) needed to be adapted.

Supplementary Table 2. ***List of reactions with relaxed $\Delta_r G'^\circ$ values (in addition to the ones shown in Supplementary Table 1) to enable growth in the iML1515_TCOSA model with measured concentration ranges.*** *Note that the $\Delta_r G'^\circ$ of the ASAD and GLUDy reaction was changed to +100 kJ to enable the reverse direction required for growth. All three reactions are reversible.*

Reaction BiGG ID	Name of the enzyme catalyzing the reaction	$\Delta_r G'^\circ$ [kJ/mol] as calculated by eQuilibrator	Corrected (relaxed) $\Delta_r G'^\circ$ [kJ/mol]
ASAD	Aspartate-semialdehyde dehydrogenase	-24.32	+100
GLUDy	Glutamate dehydrogenase (NADP)	+33.06	+100
CBMKr	Carbamate kinase	+22.07	-100

As in the main text with standard concentration ranges, we computed the (Sub)MDFs achievable for all cofactor specificity scenarios. Regarding the MDF, we found that, due to the tight concentration ranges, a single major thermodynamic bottleneck (not involving redox reactions) restricts the MDF in almost all constellations and specificity scenarios to a relatively low value of 1.9 kJ/mol (compared to 8 kJ/mol resulting from the broader standard concentration ranges used in the main text). We therefore focused our analysis on the SubMDF and found that the major findings obtained with the standard concentration ranges still hold also for the tighter (measured) concentration ranges (Supplementary Figure 2A). For example, while few random specificities reach the SubMDF of the wild-type specificity at the highest growth rate, at all lower growth rates, the SubMDF values of the wild-type specificity is advantageous over all random specificities. However, in contrast to the case with standard concentration ranges, we also found that the wild-type specificity does not fully reach the maximal SubMDF values for lower growth rates, which might be related to the very specific growth conditions and resulting metabolite concentrations associated with this dataset.

Finally, the conclusions with respect to the feasible $\frac{[NADH]/[NAD^+]}{[NADPH]/[NADP^+]}$ ratios also remain valid when the measured concentration ranges are used (Supplementary Figure 2B). The concentrations of the oxidized and reduced versions of both cofactor pools had been measured in Bennett et al.[5], but were

here, for verification, left unconstrained. We again observe tight constraints indicating that the NAD(H) pool must, in all cases, be significantly more oxidized than the NADP(H) pool (at least factor 1000) to achieve the respective SubMDF value. In fact, in all cases the $\frac{[NADH]/[NAD^+]}{[NADPH]/[NADP^+]}$ ratio must reach very small values below 0.0006. The *in vivo* value of this ratio obtained from the measured cofactor concentrations in Bennett et al.[5] is 0.00056.

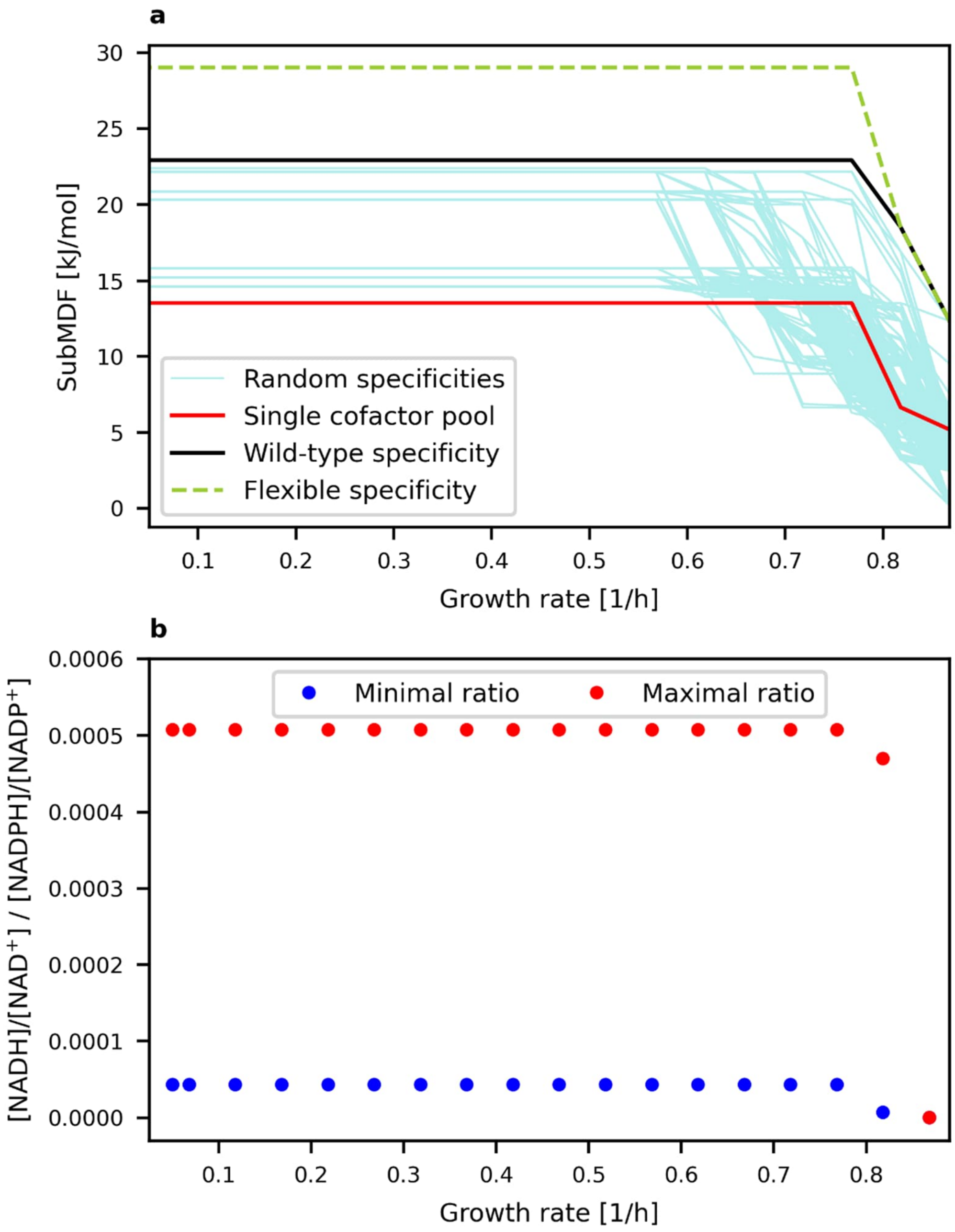

Supplementary Figure 2. ***Recalculation of key results using tight concentration ranges from Bennett et al.***[5] ***measured under aerobic conditions.*** ***a*** *SubMDF values for flexible, wild-type, and 1000 random NAD(P)(H) specificities and with a single-cofactor pool.* ***b*** *Maximal and minimal possible* $\frac{[NADH]/[NAD^+]}{[NADPH]/[NADP^+]}$ *ratios under the growth-rate-associated SubMDF value. Both results were computed for aerobic conditions to reflect the situation where the measurements have been taken. Source data are provided as a Source Data file.*

Supplementary References

1. Noor E, Bar-Even A, Flamholz A, Reznik E, Liebermeister W, Milo R. Pathway thermodynamics highlights kinetic obstacles in central metabolism. *PLOS Computational Biology* **10**, e1003483 (2014).

2. Hädicke O, Kamp Av, Aydogan T, Klamt S. OptMDFpathway: identification of metabolic pathways with maximal thermodynamic driving force and its application for analyzing the endogenous CO2 fixation potential of Escherichia coli. *PLOS Computational Biology* **14**, e1006492 (2018).

3. Salvy P, Fengos G, Ataman M, Pathier T, Soh KC, Hatzimanikatis V. pyTFA and matTFA: a Python package and a Matlab toolbox for Thermodynamics-based Flux Analysis. *Bioinformatics* **35**, 167-169 (2019).

4. Beber ME*, et al.* eQuilibrator 3.0: a database solution for thermodynamic constant estimation. *Nucleic Acids Research* **50**, D603-D609 (2022).

5. Bennett BD, Kimball EH, Gao M, Osterhout R, Van Dien SJ, Rabinowitz JD. Absolute metabolite concentrations and implied enzyme active site occupancy in Escherichia coli. *Nature Chemical Biology* **5**, 593-599 (2009).
