# Supplementary material for: Network-wide thermodynamic constraints shape NAD(P)H cofactor specificity of biochemical reactions
Source: Nat Commun. 2023 Aug 3;14:4660. doi: 10.1038/s41467-023-40297-8 (PMC10400544; doi:10.1038/s41467-023-40297-8)
Supplement: Supplementary file 3 — Description of Additional Supplementary Files [file 41467_2023_40297_MOESM3_ESM.pdf]

Description of Additional Supplementary Files

File Name: Supplementary Data 1

Description:

Sheet A: For a selected (high) growth rate (aerobic/anaerobic): Comparison of flux ranges from (loopless) flux variability Analysis and thermodynamic flux variability analysis and the maximal achievable driving force for all reactions of the central carbon metabolism or with NAD(P)(H)-dependency (all under the given MDF for this growth rate).

Sheet B: For a selected (high) growth rate (aerobic/anaerobic): Comparison of flux ranges from (loopless) flux variability analysis and thermodynamic flux Variability analysis and the maximal achievable driving force for all reactions of the central carbon metabolism or with NAD(P)(H)-dependency (all under the given SubMDF for this growth rate).

Sheet C: Aerobic concentration ranges determined by concentration variability analysis for a selected high growth rate (under its given MDF and under standard concentration ranges) and comparison with measured in vivo data from Bennett et al. (2009).

Sheet D: Aerobic concentration ranges determined by concentration variability analysis for a selected high growth rate (under its given SubMDF and under standard concentration and comparison with measured in vivo data from Bennett et al. (2009).
