# Supplementary material for: Network-wide thermodynamic constraints shape NAD(P)H cofactor specificity of biochemical reactions
Source: Nat Commun. 2023 Aug 3;14:4660. doi: 10.1038/s41467-023-40297-8 (PMC10400544; doi:10.1038/s41467-023-40297-8)
Supplement: Supplementary file 6 — Source Data [file 41467_2023_40297_MOESM6_ESM.zip › Source_Data/Readme_Source_Data.doc]

This zip folder contains all underlying Figure data of the manuscript

"Network-wide Thermodynamic Constraints Shape NAD(P)H Cofactor Specificity of Biochemical Reactions"

It is structured in subfolders named after the figures numbers.

The file names also indicate the relevant data of the (sub)figures,

starting (if possible), with the subfigure name, followed by the aerobicity,

the type of data, whether the MDF or the SubMDF is the computation target

and the used concentration ranges. "standard_concentrations" or "STANDARDCONCS"

stands for the used standard concentration ranges (see Methods), while "VIVOCONC"

stands for the used in vivo concentration ranges from Bennett et al., 2009.
